# Spontaneous Coronary Artery Dissection in a Patient With Cardiogenic Shock: To Revascularize or Not to Revascularize?

**DOI:** 10.7759/cureus.55050

**Published:** 2024-02-27

**Authors:** Kachon Lei, Brianna Yee, Michael V Dicaro, Mohamad Mubder, Omar Altaweel, Ahsan H Choudhury

**Affiliations:** 1 Cardiology, University of Nevada Las Vegas School of Medicine, Las Vegas, USA; 2 Internal Medicine, Kirk Kerkorian School of Medicine at University of Nevada Las Vegas, Las Vegas, USA; 3 Internal Medicine, University of Nevada Las Vegas School of Medicine, Las Vegas, USA; 4 Cardiology, Kirk Kerkorian School of Medicine at University of Nevada Las Vegas, Las Vegas, USA; 5 Cardiology, University Medical Center, Las Vegas, USA; 6 Cardiology, University of Nevada, Reno, USA

**Keywords:** a rare scad case required intervention, left main occlusion with cardiogenic shock, impella cp, acute coronary syndrome (acs) and stemi, scad management

## Abstract

Spontaneous coronary artery dissection (SCAD) is a rare cause of acute coronary syndrome in young patients. Supportive care is recommended for most uncomplicated cases. However, it is unclear if revascularization plays a role in treating SCAD, particularly in the setting of cardiogenic shock. We present a case of a 40-year-old female with no past medical history admitted for SCAD that was complicated by the Society for Cardiovascular Angiography & Interventions (SCAI) stage D cardiogenic shock. She was successfully managed with a percutaneous left ventricular assist device without revascularization. Repeat angiogram showed healed left anterior descending (LAD) SCAD with recovery of left ventricular (LV) systolic function. This case highlights the importance of supportive care in the treatment of SCAD, as revascularization by percutaneous coronary intervention (PCI) and coronary artery bypass graft surgery (CABG) can pose a significant perioperative risk in this patient population.

## Introduction

Spontaneous coronary artery dissection (SCAD), once considered a rare cause of acute coronary syndrome (ACS), is now increasingly identified and acknowledged as a significant clinical entity [1]. SCAD arises from the spontaneous, non-traumatic separation of the coronary arterial wall, typically due to an intramural hematoma (IMH) caused by an intimal tear or spontaneous hemorrhage from the vaso-vasorum [2]. The widespread use of coronary angiography, coupled with the enhanced clinical utility of intra-coronary imaging techniques like optical coherence tomography (OCT) and intravascular ultrasound (IVUS), has led to greater awareness of this condition [3]. Despite these advancements, SCAD is frequently overlooked and misdiagnosed due to its similar appearance in coronary angiography to atherosclerotic coronary artery disease. Maintaining a high clinical suspicion, particularly in young patients, is crucial for accurate diagnosis [3]. 

Once the diagnosis is made, clinical management has been primarily supportive care in uncomplicated cases without ongoing ischemia and hemodynamic instability. However, there is a lack of data on how to manage SCAD patients with cardiogenic shock. Some suggest revascularization with percutaneous coronary intervention (PCI) or coronary artery bypass graft surgery (CABG) may improve patient outcomes. However, no randomized data supports using PCI and CABG in such scenarios. We herein present a case report of SCAD that is complicated by cardiogenic shock, which we successfully managed with supportive care alone with a percutaneous left ventricular assist device (Impella CP; Johnson & Johnson, New Brunswick, New Jersey). We propose that hemodynamic support should be the mainstay management of SCAD patients presenting with cardiogenic shock to avoid complications from PCI and CABG surgery.

## Case presentation

**Figure 1:**
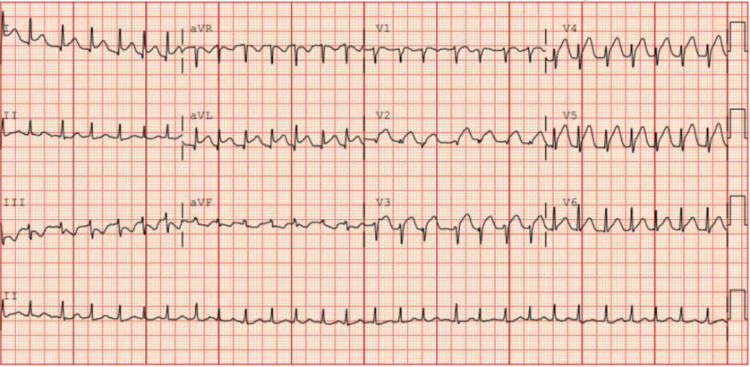
Initial EKG in the emergency department revealing ST elevations in 1, aVL, and V2-V5

**Figure 2:**
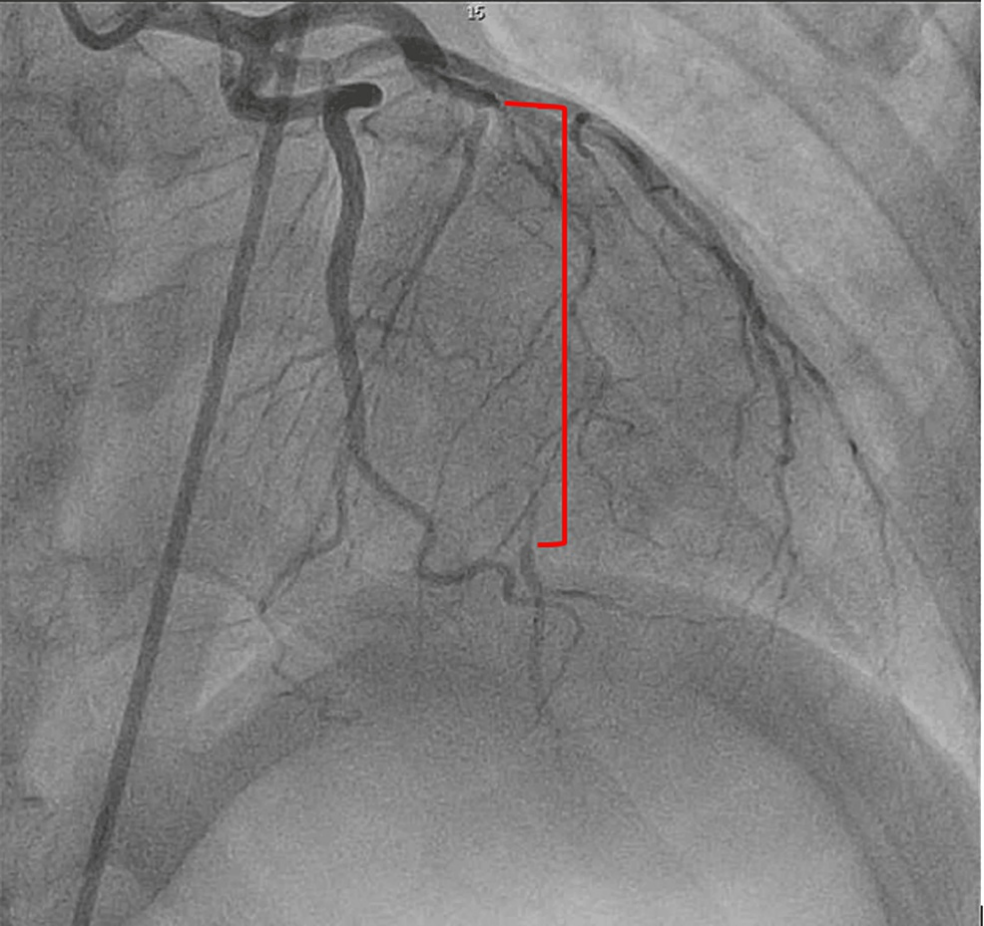
Spontaneous coronary artery dissection (red bracket) in the left anterior descending artery, measuring approximately 40 mm in length

**Figure 3:**
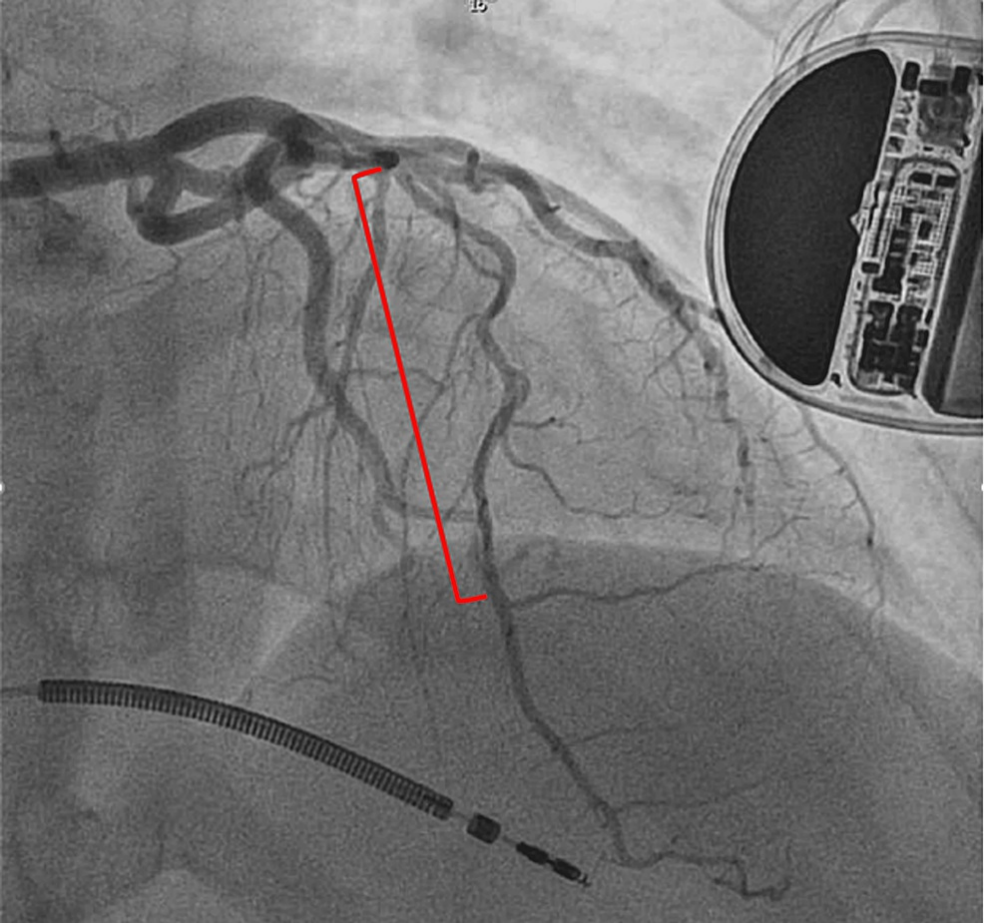
Repeat angiogram one week later, showing healed LAD artery (red bracket) LAD - left anterior descending

A 40-year-old woman with no known past medical history presented to the emergency department with substernal chest pain radiating to both arms and associated vomiting. Upon arrival at the emergency department, she was seen in severe pain. The patient was tachycardic at 170 bpm with a blood pressure of 154/90, with low oxygen saturation at 85% on room air. Initial EKG showed ST-segment elevations in leads 1, aVL, V2-5 (Figure 1). Shortly after, she was found to be in ventricular fibrillation. Advanced cardiac life support (ACLS) was initiated with a return of spontaneous circulation after epinephrine and shock. Lactic acid was 9.71 mmol/L, and Cr was 1.81 mg/dl. Troponin was elevated at 20,303 ng/L. She was intubated in the ED, and she was taken for immediate left heart catheterization (LHC). A coronary angiogram revealed type 2 SCAD originating from the mid to distal left anterior descending artery (Figure 2), approximately 40mm long. The left ventriculogram showed an ejection fraction of 30%, with no evidence of mitral regurgitation. Right heart catheterization revealed a normal pulmonary artery pulsatility index at 2.1, elevated mean pulmonary artery (PA) pressure of 35 mmHg, and low PA oxygen saturation of 58%. Wedge pressure was elevated at 30 mmHg, and cardiac output was normal at 5.09 L/min with a cardiac index of 2.54 while on inotropic support. Due to evidence for cardiogenic shock, in addition to profound hypoxia, escalating vasopressor requirement, and unstable cardiac rhythm, the decision was made to place an Impella CP for hemodynamic support. CT surgery was consulted, and they recommended supportive care. The patient was subsequently admitted to the CCU on aspirin and ticagrelor without revascularization. She was weaned off vasopressor support within 12 hours, and the Impella was discontinued on hospital day (HD) two. She underwent ICD placement for ventricular fibrillatory cardiac arrest. On HD seven, the patient underwent a repeat LHC, which displayed a healed dissection in the LAD (Figure 3). She was discharged in stable condition with aspirin, ticagrelor, and metoprolol. She was instructed to follow up with cardiology outpatient after discharge with evaluation for fibromuscular dysplasia.

## Discussion

SCAD is characterized as a coronary artery dissection affecting the epicardial vessels unrelated to atherosclerosis, trauma, or iatrogenic causes. The majority of cases manifest as symptoms of acute coronary syndrome [4]. Although previously considered an uncommon cause of myocardial infarction, SCAD is now recognized as a significant factor, particularly among young to middle-aged women, commonly found in patients with fibromuscular dysplasia and other connective tissue diseases. Maintaining a heightened clinical suspicion is crucial, especially for young female patients experiencing chest pain without traditional risk factors [3]. In highly suspicious cases, coronary angiography is typically the initial diagnostic step for SCAD. Non-invasive imaging studies, such as coronary CT scans, cardiac MRI, and myocardial perfusion imaging, may also be considered in specific situations [5].

Due to a lack of randomized trials comparing medical therapy and revascularization strategies, there is limited data on the optimal management of SCAD. Observational data, however, suggest that conservatively managed patients often exhibit angiographic healing of SCAD lesions upon repeat angiography, ranging from 70-97% [6,7]. In most cases, a conservative approach is recommended by society guidelines, involving an extended hospital stay for close monitoring. Long-term medical therapy, primarily consisting of aspirin and a beta-blocker, is advised [8]. Despite the evidence on supportive care, data suggest that revascularization may play a role in treating SCAD. Current literature indicates that revascularization can be considered in cases with active myocardial ischemia and hemodynamic instability, where conservative management is deemed unsatisfactory. CABG surgery is reserved for patients with left main or multi-vessel proximal large-vessel dissection [3]. Interestingly, Tweet et al. demonstrated similar outcomes (death, recurrent SCAD, heart failure, target vessel revascularization, and left ventricular ejection fraction) comparing CABG vs. PCI vs conservative medical therapy, even in the setting of left main artery dissection with the presence of cardiogenic shock (p=0.12). Little evidence supports that revascularization is superior to the high-risk coronary dissection group. As of today, the decision on revascularization has been an expert opinion, determined on a case-by-case basis. Furthermore, PCI in SCAD patients was associated with significantly higher rates of complications, including PCI failure (up to 50%) and the need for emergency CABG bailout (up to 13%) [9]. Unfortunately, PCI in SCAD is technically challenging, as guide wires can propagate vessel dissection and perforation. Stent struts can also cause hematoma propagation and late strut malapposition as the hematoma gets resorbed, causing late stent thrombosis [9]. In addition, bypass graft conduit occlusion due to competitive flow has been reported in SCAD patients who underwent CABG surgery in five years follow-up [9]. This evidence further supports our theory that the majority of SCAD patients with cardiogenic shock, therefore, should be managed conservatively with hemodynamic support instead of emergency revascularization. Importantly, the literature demonstrates that high-risk coronary anatomy, such as left main dissection, can be safely managed with mechanical circulatory support such as Impella or venoarterial extracorporeal membrane oxygenation (VA-ECMO) as a bridge to recovery in this patient population [10]. 

## Conclusions

In conclusion, our case supports previous findings of self-limited healing of SCAD. Although our patient presented in critical condition requiring CCU admission, mechanical support, and vasopressor therapy, our conservative approach to the management of SCAD proved to be sufficient. She was monitored closely in the hospital, and repeat angiography confirmed the resolution of her dissection despite our non-aggressive approach. We highlight this case to provide additional information on the infrequent occurrence of SCAD and insights on recommended therapy and outcomes. Further studies will be needed to elucidate this specific patient population's most optimal treatment modality.

## References

[1] Franke KB, Wong DT, Baumann A, Nicholls SJ, Gulati R, Psaltis PJ (2019). Current state-of-play in spontaneous coronary artery dissection. Cardiovasc Diagn Ther.

[2] Saw J, Starovoytov A, Aymong E (2022). Canadian spontaneous coronary artery dissection cohort study: 3-year outcomes. J Am Coll Cardiol.

[3] Boulmpou A, Kassimis G, Zioutas D (2020). Spontaneous coronary artery dissection (SCAD): case series and mini review. Cardiovasc Revasc Med.

[4] Britel D, Nikièma S, Massimbo D, Graham E, Benyass A, Lakhal Z (2023). Spontaneous coronary artery dissection (SCAD): a case report. Ann Med Surg (Lond).

[5] Krittanawong C, Saw J, Olin JW (2020). Updates in spontaneous coronary artery dissection. Curr Cardiol Rep.

[6] Rashid HN, Wong DT, Wijesekera H (2016). Incidence and characterisation of spontaneous coronary artery dissection as a cause of acute coronary syndrome - a single-centre Australian experience. Int J Cardiol.

[7] Rogowski S, Maeder MT, Weilenmann D (2017). Spontaneous coronary artery dissection: angiographic follow-up and long-term clinical outcome in a predominantly medically treated population. Catheter Cardiovasc Interv.

[8] Saw J, Mancini GB, Humphries KH (2016). Contemporary review on spontaneous coronary artery dissection. J Am Coll Cardiol.

[9] Krittanawong C, Gulati R, Eitzman D, Jneid H (2021). Revascularization in patients with spontaneous coronary artery dissection: where are we now?. J Am Heart Assoc.

[10] Zanchi J, Miric D, Giunio L (2022). Conservative management of spontaneous left main coronary artery dissection (SCAD) triggered by emotional stress in the late postpartum period: case report and pathophysiology. Pathophysiology.

